# The association between satisfaction with life and anxiety symptoms among Chinese elderly: a moderated mediation analysis

**DOI:** 10.1186/s12877-023-04490-0

**Published:** 2023-12-14

**Authors:** Cynthia Y. Y. Lai, Lu Hua Chen, Frank H. Y. Lai, Ada W. T. Fung, Shamay S. M. Ng

**Affiliations:** 1 Department of Rehabilitation Sciences, Faculty of Health and Social Sciences, The Hong Kong Polytechnic University, Hong Kong SAR, China; 2 Research Centre for SHARP Vision (RCSV), The Hong Kong Polytechnic University, Hong Kong SAR, China; 3 Research Institute for Smart Ageing (RISA), The Hong Kong Polytechnic University, Hong Kong SAR, China; 4 Mental Health Research Center (MHRC), The Hong Kong Polytechnic University, Hong Kong SAR, China; 5 Department of Social Work, Education and Community Wellbeing, Faculty of Health and Life Sciences, The Northumbria University, Newcastle upon Tyne, UK; 6 Department of Sport, Physical Education and Health, Faculty of Social Sciences, Hong Kong Baptist University, Hong Kong SAR, China

**Keywords:** Satisfaction with life, Anxiety symptoms, Cognitive function, Chinese elderly

## Abstract

**Background:**

Previous studies have suggested that certain personal psychological variables (e.g., life satisfaction and cognitive function) and physical variables (e.g., body mass index [BMI]) are significantly associated with individuals’ anxiety symptoms. However, relevant research on elderly is lagging and no studies have yet investigated the combined impact of these variables on anxiety. Thus, we conducted the present study to investigate the potential moderator role of BMI and the potential mediator role of cognitive function underlying the relationship between life satisfaction and anxiety symptoms in Chinese elderly based in Hong Kong.

**Methods:**

Sixty-seven elderly aged 65 years old and above were recruited from the local elderly community centres in this pilot study. Each participant underwent a systematic evaluation using the Satisfaction with Life Scale (SWLS), Hong Kong Version of the Montreal Cognitive Assessment (HK-MoCA), and the Hamilton Anxiety Rating Scale (HAM-A) and were measured for their body weight and height. Regression analysis using the bootstrapping method was employed to test the hypothesized moderated mediation model.

**Results:**

Our findings demonstrated the overall model accounted for 23.05% of the variance in scores of HAM-A (F (8, 57) = 2.134, *p* = 0.047) in Chinese elderly. There was a significant association between life satisfaction and anxiety symptoms (*p* = 0.031), indicating that individuals with higher life satisfaction were associated with less anxiety symptoms. Moreover, this relationship was positively moderated by BMI (*b* = 0.066, 95% CI [0.004, 0.128]), especially in Chinese elderly with BMI at a lower level (*b* = -0.571, 95% CI [-0.919, -0.224]) and an average level (*b* = -0.242, 95% CI [-0.460, -0.023]). No significant mediator role was detected for cognitive function (*b* = -0.006, 95% CI [-0.047, 0.044]) in our model.

**Conclusions:**

Our findings suggest that increased life satisfaction can reduce anxiety symptoms among Chinese elderly as their BMI decreases (when BMI ranged between “mean - 1SD” and “mean” of the population). The significant interaction between psychological and physical factors underlying anxiety symptoms found in this study, presents a promising opportunity for translation into multi-level psychological and physical interventions for the management of anxiety in ageing patients during clinical practice.

## Background

Anxiety is an unpleasant emotion (e.g., a feeling of fear or worry about an anticipated event) accompanied by somatic complaints. It is a common mental health problem. According to epidemiological data from Western countries, the lifetime prevalence of anxiety disorder ranges from 14.5% to 33.7% in the general population [1–3]. Although anxiety disorder is a widespread mental health concern, its prevalence among elderly has only attracted more scientific interest in recent decades. In the community-dwelling elderly, it has been reported that the prevalence of anxiety disorder varies from 1.2% to 17.2% [4–6], while the estimated prevalence for elderly in clinical settings is higher at 28% [7]. Differing from clinical diagnosed anxiety disorder, the prevalence of anxiety symptoms that do not satisfy the criteria for clinically diagnosed anxiety disorder is estimated to be 15.0%–56.0% in elderly [7]. A national survey of the general population in China revealed a lifetime prevalence for anxiety disorder of 7.6% [8]. For Chinese elderly, the prevalence of anxiety disorder is 3.71%, according to a recent study conducted by Xu et al. [9]. A review of the prevalence of anxiety symptoms among more specific populations of Chinese elderly showed a prevalence of 7.9%–20.8% for those dwelling in communities [10–12], and 17.2%–21.1% for those in primary care settings [13]. Some environmental and psychological factors have been reported to be associated with anxiety in the elderly, such as females, poor health status, poor economic situations, feelings of loneliness, lower level of social trust and participation, etc. [12]. Anxiety in later life has impactful consequences, as it is associated with increased risks of cognitive impairment [14], cardiovascular comorbidity [15], a lower quality of life [16], and severe disabilities [17], all of which lead to heavy burdens on health services. Therefore, investigating the early stages of anxiety in elderly (e.g., anxiety symptoms at the subclinical level) to identify other contributing factors and underlying neuropathological mechanisms are helpful in determining early therapeutic interventions for this specific population.

One of the contributing factors to anxiety is satisfaction with life (SWL), which is a subjective judgement of an individual’s quality of life involving a comparative process based on their own life standards [18]. It reflects an individual’s psychological well-being through a measurement of the degree of agreement between their current life, their expected life, and their achieved life. Recently, increasing studies have examined the critical role of SWL in predicting mental health problems [19–25]. Fergusson et al. investigated the associations between SWL and a number of psychiatric disorders, including depression, anxiety, suicidality, alcohol dependence, and drug addiction based on a birth cohort study in New Zealand. In addition to significant associations with depression, suicidality, and drug addiction, they found significant associations between SWL and anxiety disorder using a fixed effects model regression analysis. Further examination of the direction of causality between SWL and mental health problems using a structural equation model revealed robust and reciprocal associations [19]. Differing from Fergusson et al.’s study targeting on psychiatry disorders which needs participants fulfilled in clinical diagnostic criteria of mental disorder, in another study based on a national community survey carried out in Canada, Lombardo et al. examined the associations between SWL and self-reported mental health status of anxiety/mood disorders. Consistently, they found that SWL is associated with self-reported anxiety/mood disorders and that lower SWL is associated with self-reports of poor mental health status [22]. However, these previous studies were mainly carried out in adolescent and young adult populations, with the elderly population been overlooked.

In addition to SWL, previous research has suggested that impaired cognitive function is another contributing factor to anxiety. Mantella et al. found that older patients with clinically diagnosed anxiety disorder had impaired cognitive function in short-term memory [26]. In another study conducted by Butters et al., older patients diagnosed with anxiety disorder had impaired cognitive abilities in multiple domains, including memory, inhibition, information processing, and problem-solving [27]. The impactful associations with multiple cognitive domains are not only present in individuals with clinically diagnosed anxiety, but also present in individuals with anxiety symptoms at subclinical levels [28, 29]. Consistent with the findings demonstrated in cross-sectional studies, findings from longitudinal studies have also demonstrated positive associations between the severity of anxiety (both clinically diagnosed disorders and symptoms at subclinical levels) and the incidence of cognitive impairment in elderly [30]. Because of the limited number of longitudinal studies investigating the associations, the direction of causality between impaired cognitive function and anxiety is not completely understood. Beaudreau et al. proposed a bidirectional relationship between these two factors [31]. In this bidirectional model, anxiety and cognitive impairment exacerbate each other’s risks and symptoms, with anxiety leading to later cognitive impairment and pre-existing cognitive impairment leading to increased levels of anxiety.

Body mass index (BMI) is calculated as an individual’s weight in kilograms divided by their height in metres squared (kg/m^2^), and is commonly used to assess physical health with respect to obesity [32]. There are four BMI classifications: underweight (≤ 18.4 kg/m^2^), normal weight (≥ 18.5 and ≤ 24.9 kg/m^2^), overweight (≥ 25.0 and ≤ 29.9 kg/m^2^), and obese (≥ 30.0 kg/m^2^). An increasing number of studies have suggested associations between unfavourable BMI (e.g., overweight and obese) and a number of chronic physical diseases, including diabetes, hypertension, coronary heart disease, stroke, and cancer [33, 34]. Furthermore, there is an accumulation of evidence showing the contribution of an unfavourable BMI to the development of anxiety and other mental health problems, e.g., mood and alcohol disorders [35]. Leonore et al. conducted a longitudinal cohort study in the Netherlands and found that obese individuals have an increased risk of developing anxiety [36]. In this prospective study which included 5303 participants, compared to individual with normal BMI, obese individual at baseline were found to have a significantly higher risk of onset of anxiety disorder during follow-up (odds ratio = 1.71). The positive association between unfavourable BMI and anxiety was further confirmed by a systematic review and meta-analysis. In this study, Gariepy et al. included 16 studies (2 prospective studies and 14 cross-sectional studies) and revealed a pooled odds ratio of 1.4 (with 95% confidence interval of 1.20–1.60) for association between obesity and anxiety disorder [37]. Consistently, in another systematic review and meta-analysis performed by Amiri et al., findings indicated a pooled odds ratio of 1.1 (with 95% confidence interval of 1.20–1.41) for anxiety symptoms in overweight individuals, and a pooled odds ratio of 1.3 (with 95% confidence interval of 1.00–1.21) for anxiety symptoms in obese individuals [38]. Interestingly, in a study based on data from 103,557 individuals aged 18–85 years old in the United States, a U-shaped association was demonstrated, suggesting that individuals who are either underweight or overweight have higher risks of developing anxiety than individuals with normal BMI [39]. However, the majority of studies investigating the impact of BMI on anxiety have been carried out in Western countries and studies of Chinese and in particular, older Chinese adult populations, remain scarce.

The psychological variables, SWL and cognitive function, and the physical variable, BMI, have been reported to be associated with anxiety, but relevant research in elderly is lagging and no studies have yet investigated the combined impact of these variables on anxiety symptoms at subclinical levels. Therefore, the aim of the present study was to investigate the impact of SWL on anxiety symptoms in Chinese elderly based in Hong Kong. We speculated a potential moderator role of BMI and a potential mediator role of cognitive function underlying the relationship between SWL and anxiety symptoms. We hypothesized that among older adults in Hong Kong: 1) SWL is negatively associated with anxiety symptoms; 2) the negative association between SWL and anxiety symptoms is moderated by BMI; 3) the negative association between SWL and anxiety symptoms is partially mediated by the cognitive function of elderly.

## Methods

### Participants

In this cross-sectional cohort study, participants were recruited from local communities, e.g., Neighbourhood Elderly Centre in Hong Kong. Participants were included if they were: (1) aged 65 years old or above; (2) able to speak Cantonese; and (3) ambulatory. Participants were excluded if they: (1) had uncorrected visual or auditory impairment; (2) were unable to follow assessment instructions from the research personnel; or (3) had any non-psychiatric chronic medical conditions (e.g., chronic kidney disease, or diabetes). The Hong Kong Polytechnic University Institutional Review Board approved the present study. Sample size calculation was conducted using G*power for a multiple regression analysis [40] as in a previous study [41]. The parameters for power analysis were set as: *f*^*2*^ = 0.15 (medium effect size), a power of 0.80, and an α = 0.05. A minimum of 55 participants were required to detect the medium effect size for the proposed 4 variables with 80% power.

### Procedures

Prior to the study, a research package with an information sheet and consent form were given to each participant. The research personnel explained the objectives and procedures of the study to each participant and obtained their written informed consent. Each participant was then requested to provide demographic information, including their age, gender, education, and medical history, in addition to their body weight and height measurements. Subsequently, each participant was tested for their intelligence level using the Test of Nonverbal Intelligence, Fourth Edition (TONI-4). Finally, life satisfaction, cognitive function, and anxiety symptoms, were systematically evaluated using the respective instruments: the Satisfaction with Life Scale (SWLS), Hong Kong Version of the Montreal Cognitive Assessment (HK-MoCA), and the Hamilton Anxiety Rating Scale (HAM-A). All assessments were performed through face-to-face conversations between the participants and the research personnel, with frequent rest periods offered to avoid mental fatigue.

### Instruments

#### Satisfaction with Life Scale (SWLS)

The SWLS was employed to evaluate participants’ subjective feelings of life satisfaction [42]. It is a five-item survey with a 7-point Likert response scale (from ‘strongly disagree’ to ‘strongly agree’). Total scores for the SWLS range from 5 to 35, with lower scores indicating more dissatisfaction with life and higher scores indicating more satisfaction with life [43]. The test–retest reliability of the SWLS have been confirmed by others (test–retest correlation coefficient = 0.85), which demonstrates that it is suitable to be used in the elderly and is reliable in determining affect status [44]. The Chinese version of SWLS has consistently shown high internal consistency (Cronbach's alpha = 0.91), and has been widely used [45].

#### Hong Kong version of the Montreal Cognitive Assessment (HK-MoCA)

The Montreal Cognitive Assessment is a widely used assessment tool for cognitive function in older individuals. It consists of seven components including 1) Attention/Concentration, 2) Naming, 3) Executive/Visuospatial Function, 4) Language, 5) Delayed Recall, 6) Orientation, and 7) Abstract Reasoning. Previous studies have shown that the assessment has good internal consistency (Cronbach's alpha = 0.83) and test–retest reliability (test–retest correlation coefficient = 0.92) [46]. The HK-MoCA has been translated from English to Cantonese with appropriate cultural and linguistic modifications [47] and has been validated in older Chinese adults with mild cognitive impairment and dementia (with a sensitivity of 0.92 as well as specificity of 0.92) [48]. The overall scores for the HK-MoCA range from 0 to 30. Assessment scores were calculated by adding the sub-scores of the seven components. Higher scores indicate better cognitive function.

#### Hamilton Anxiety Rating Scale (HAM-A)

The HAM-A is a widely used interview scale in research and clinical settings for the measurement of the severity of anxiety symptoms in older individuals. This scale has fourteen items measuring anxiety symptoms from multiple dimensions, including tension, anxious mood, insomnia, fears, intellectual, depressed mood, somatic complaints (e.g., muscular, sensory, cardiovascular, respiratory, gastrointestinal, and genitourinary symptoms), and behaviour at interview. It uses a 5-point Likert response scale (from ‘not present’ to ‘severe’). Total scores range from 0 to 56, with higher scores suggesting more severe anxiety symptoms in a participant. The test–retest reliability of the HAM-A have been confirmed by others (test–retest correlation coefficient = 0.74) [49]. The Chinese version of HAM-A has also shown good internal consistency (Cronbach's alpha = 0.80), and is regarded as a reliable tool for measurement of anxiety symptoms in Chinese outpatients [50].

#### Test of Nonverbal Intelligence, Fourth Edition (TONI-4)

The TONI-4 is a test that aims to assess an individual’s general intellectual functioning, e.g., abilities for problem solving and abstract reasoning, which is relatively consistent over an individual’s lifetime. This assessment tool has demonstrated good test–retest reliability (test–retest correlation coefficient = 0.93) in Chinese and can be used in individuals aged from 6 to 89 years old [51]. The benefit of TONI-4 is its language-free format. As the results of the TONI-4 are not influenced by an individual’s hearing or linguistic capacity, it is especially appropriate for elderly who often have hearing or linguistic deficiencies. Form A of the test consists of five training items, and forty-five problem solving items were used in the present study [52]. Each problem-solving item has a sequence of abstract figures (for demonstration) as well as a missing figure which needs participant’s responses. Difficulty of each problem-solving item is presented in an ascending order. The correct answer for each item was awarded with one point. The overall score, which was the sum of the score for the forty-five items, was then converted into an index score for subsequent data analysis. Higher index score indicates better problem-solving and abstract reasoning capacities of the participant.

### Statistical analyses

Data analyses were performed using Statistical Package for the Social Sciences version 23 (SPSS v.23; IBM, Armonk, NY, USA). Pearson’s correlation analysis was performed to test the relationships among the variables of interest, including BMI, life satisfaction, cognitive function, and symptoms of anxiety. Subsequently, multiple linear regression was conducted to investigate the specific associations of BMI, life satisfaction, and cognitive function with symptoms of anxiety, separately, Adjustments for age, gender, education level, and TONI-4 index score were included in the multiple linear regression model to exclude any confounding effects. Finally, moderated mediation analysis testing the hypothesized moderator and mediator was run using the bootstrapping principle by applying the Hayes’s PROCESS macro programme [53]. This analysis involves a nonparametric procedure that produces a confidence interval (CI) by integrating the Syntax programme in SPSS. The potential moderating effect of BMI and the potential mediating effect of cognitive function were evaluated using PROCESS macro programme Model 5, based on 10,000 bootstrapping resampling events after adjustment for age, gender, education level, and TONI-4 index score. All continuous variables included in the model were mean centred. For the moderated mediation analysis, if zero wasn’t included in the calculated CI, the moderating effect or the mediating effect was considered to be significant. For the rest of the data analyses, *p* < 0.05 was considered to indicate statistical significance.

## Results

### Participants’ demographic characteristics

As shown in Table 1, total 67 elderly with a mean age of 70.96 ± 5.04, were recruited in the present study, including 56 females (83.6%) and 11 males (16.4%). Of these 67 elderlies, 10.4% had an educational attainment below primary school and 89.6% had an educational attainment of primary school or higher. The mean BMI of the participants was 23.72 ± 5.05 kg/m^2^. The mean TONI-4 index, SWLS, HK-MoCA, and HAM-A scores were, 100.25 ± 10.33, 27.00 ± 5.40, 25.21 ± 3.59, and 6.09 ± 4.81, respectively.

**Table 1:** Demographic characteristics of all elderly

	**All participants **(***n*** = 67)
Age, years (mean ± SD)	70.96 ± 5.04
Gender	
Male (n, %)	11 (16.4%)
Female (n, %)	56 (83.6%)
Education level	
Below primary school (n, %)	7 (10.4%)
Primary school (n, %)	31 (46.3%)
Secondary school (n, %)	18 (26.9%)
Post-secondary degree (n, %)	3 (4.5%)
Bachelor degree or above (n, %)	8 (11.9%)
Body weight (mean ± SD) (kg)	56.36 ± 12.62
Body height (mean ± SD) (m^2)^	1.54 ± 0.07
BMI (mean ± SD)	23.72 ± 5.05
TONI-4 index score (mean ± SD)	100.25 ± 10.33
SWLS score (mean ± SD)	27.00 ± 5.40
HK-MoCA score (mean ± SD)	25.21 ± 3.59
HAM-A score (mean ± SD)	6.09 ± 4.81

*BMI* Body Mass Index, *TONI-4* Test of Nonverbal Intelligence, Fourth Edition, *SWLS* Satisfaction with Life Scale, *HK-MoCA* Hong Kong Version of Montreal Cognitive Assessment, *HAM* Hamilton Anxiety Rating Scale

### Pearson’s correlation analysis

Table 2 shows the Pearson’s correlation coefficients for the variables of interest. The results show that SWLS scores were negatively correlated with HAM-A scores (*r* = -0.338, *p* = 0.005), indicating that a higher level of life satisfaction was associated with less anxiety symptoms in the study population (Table 2).

**Table 2:** Pearson correlation analysis among studied variables

	**BMI**	**SWLS**	**HK-MoCA**	**HAM-A**
BMI	1	0.161	-0.118	-0.123
SWLS	0.161	1	-0.187	-0.338*
HK-MoCA	-0.118	-0.187	1	0.137
HAM-A	-0.123	-0.338*	0.137	1

*BMI* Body Mass Index, *SWLS* Satisfaction with Life Scale, *HK-MoCA* Montreal Cognitive Assessment Hong Kong version, *HAM* Hamilton Anxiety Rating Scale

^*^P < 0.01

### Moderated mediation analysis

**Fig. 1:**
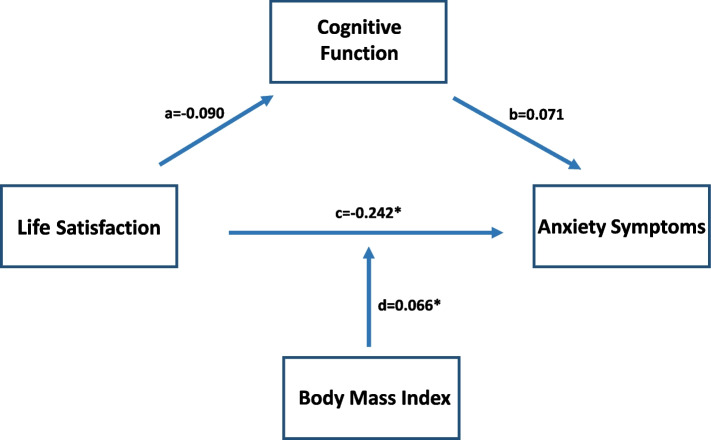
The moderated mediation model for life satisfaction on symptoms of anxiety in Chinese elderly Significances were found for the direct effect of life satisfaction on symptoms of anxiety (*p* = 0.031) (path c) and moderating effect of BMI (*p* = 0.038) (path d) after adjustment for age, gender, education level, and TONI-4 index scores. However, no significant indirect effect was detected for cognitive function as proposed in our model (*b* = -0.006, 95% CI [-0.047, 0.044]) (path a*b). The overall moderated mediation model accounting for 23.05% of the variance in scores of HAM-A (*F* (8, 57) = 2.134, *p* = 0.047). *Significant at *p* < 0.05

**Fig. 2:**
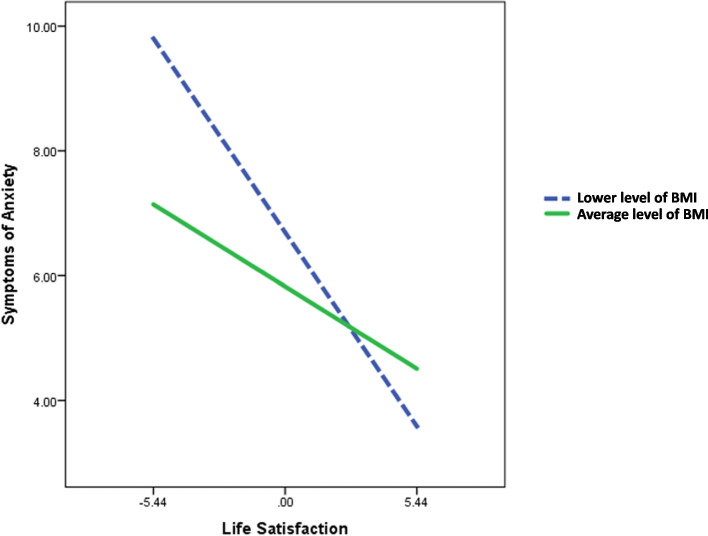
The moderating effect of BMI on the relationship between life satisfaction and symptoms of anxiety in Chinese elderly

Consistent with the findings of the Pearson’s correlation analysis, SWLS scores remained significantly associated with HAM-A scores after adjustment for age, gender, education level, and TONI-4 index score (*b* = -0.242, *p* = 0.031). There was no significant association between HK-MoCA scores and HAM-A scores after adjustment for age, gender, education level, and TONI-4 index score. No significant association was identified between BMI and HAM-A scores (Fig. 1). However, when taken as a set, the proposed moderated mediation model accounted for 23.05% of the variance in HAM-A scores (*F* (8, 57) = 2.134, *p* = 0.047). Moreover, we found a significant interaction between SWLS scores and BMI (*F* (1, 57) = 4.525, *p* = 0.038) among Chinese elderly. A significant conditional direct effect of SWLS scores on HAM-A scores was identified at different levels of BMI (presented as the mean – 1 SD, mean, and mean + 1 SD; Table 3). The direct effect of life satisfaction on symptoms of anxiety was significant for Chinese elderly with a lower level of BMI (mean – 1 SD; *b* = -0.571, 95% CI [-0.919, -0.224]) and an average level of BMI (mean; *b* = -0.242, 95% CI [-0.460, -0.023]), which indicates stronger association between life satisfaction and symptoms of anxiety as BMI value decreases (Fig. 2). Whereas for elderly with higher level of BMI (mean + 1 SD), the direct effect of life satisfaction on the symptoms of anxiety was not significant (*b* = 0.088, 95% CI [-0.321, 0.497]). Subsequent John–Neyman analysis showed that the impact of life satisfaction on the symptoms of anxiety was significantly moderated by BMI, until a value of 24.12 kg/m^2^ (*p* = 0.05; 95% CI [-0.445, 0.000]; Table 4). No significant indirect mediating effect was detected for cognitive function, as proposed in our model (*b* = -0.006, 95% CI [-0.047, 0.044]).

**Table 3:** Conditional direct effects of life satisfaction on symptoms of anxiety at different BMI level

	**Effect**		**95% CI**	
	***b***	***t***	**Lower**	**Upper**
**Low BMI (mean - 1SD)**	-0.571	-3.294	-0.919	-0.224
**Average BMI (mean)**	-0.242	-2.218	-0.460	-0.023
**High BMI (mean + 1SD)**	0.088	0.430	-0.321	0.497

*b* Unstandardized Coefficient, *CI* Confidence Interval

Adjustment for age, gender, education level, and TONI-4 index score

**Table 4:** Specific BMI values defining Johnson-Neyman significance regions

BMI value (kg/m^2^)	Effect		95% CI	
	***b***	***t***	**Lower**	**Upper**
9.800	-1.164	-2.717	-2.022	-0.306
11.230	-1.070	-2.773	-1.842	-0.297
12.659	-0.976	-2.841	-1.664	-0.288
14.089	-0.882	-2.921	-1.486	-1.486
15.518	-0.788	-3.018	-1.311	-0.265
16.948	-0.694	-3.132	-1.138	-0.250
18.377	-0.600	-3.258	-0.969	-0.231
19.807	-0.506	-3.362	-0.807	-0.205
21.236	-0.412	-3.332	-0.660	-0.164
22.666	-0.318	-2.923	-0.536	-0.100
24.095	-0.224	-2.021	-0.446	-0.002
24.121	-0.222	-2.003	-0.445	0.000
25.525	-0.130	-1.008	-0.388	0.128
26.954	-0.036	-0.228	-0.352	0.280
28.384	0.058	0.301	-0.327	0.443
29.813	0.152	0.660	-0.310	0.614
31.243	0.246	0.910	-0.295	0.787
32.672	0.340	1.092	-0.284	0.964
34.102	0.434	1.229	-0.273	1.141
35.531	0.528	1.335	-0.264	1.320
36.961	0.622	1.419	-0.256	1.499
38.390	0.716	1.488	-0.248	1.679

*b* Unstandardized Coefficient, *CI* Confidence Interval

Adjustment for age, gender, education level, and TONI-4 index score

## Discussion

In the present study, we examined the association between SWL and anxiety symptoms among Chinese elderly and its potential underlying mechanisms. Our findings showed that SWL was negatively associated with anxiety symptoms (Hypothesis 1), and this effect was moderated by BMI (Hypothesis 2). However, cognitive function was not a mediator of the relationship between SWL and anxiety symptoms among Chinese elderly as revealed by the present study (Hypothesis 3).

Consistent with previous findings [19, 21–24], we found that a higher SWL was associated with less severe symptoms of anxiety among Chinese elderly. SWL, which reflects a positive life perception, has been suggested to play an important role in maintaining subjective well-being [54]. Subjective well-being, which is conceptualised as positive psychology, is a recently established discipline that emphasises taking a positive perspective on things [55]. It has been suggested that positive affect and cognitive processes are powerful protective factors against worry and can buffer dysfunctional stress and thoughts that may lead to anxiety [56–58]. Conversely, dissatisfaction with life, which reflects negative perceptions of life, has been documented to predict anxiety across an individual’s lifespan [59]. In later adulthood, individuals are often faced with unfavourable life changes related to decreased life satisfaction, such as fewer social connections, the loss of partners, and financial dependence. These adverse life changes may result in feelings of loneliness, helplessness, and uselessness, which lead to negative thoughts and affect. Without appropriate interventions, negative life perception, accompanied by chronic worry and stress, may gradually cause anxiety symptoms in older adults. The current evidence supporting the protective role of higher SWL against anxiety symptoms in Chinese elderly has important clinical implications and the potential for being translated into an intervention using positive psychology. Psychological interventions focusing on the positive side of things increase perceptions of pleasure, satisfaction, and well-being, and have demonstrated significant clinical effectiveness. In a recent meta-analysis, Chakhssi et al. demonstrated a moderate effect size for utilising positive psychology to induce anxiety remission [60]. In light of all of the above evidence, it is time to promote an appropriate positive psychology programme to increase life satisfaction to prevent anxiety symptoms in Chinese elderly.

Additionally, our findings have shown that the neuroprotective effect of SWL on anxiety symptoms is moderated by BMI (a physical factor). For Chinese elderly with lower and average levels of BMI, a higher SWL had a protective effect on anxiety symptoms, whereas, for those with a higher level of BMI, the significant impact of SWL on anxiety symptoms disappeared. The cut-off for a higher level of BMI in the present study was 24.12 kg/m^2^ (Table 4), which approximately represents overweight and obesity. It has been suggested that individuals with an elevated BMI, particularly those with obesity, have elevated concentrations of serum inflammatory markers, such as C-reactive protein, tumour necrosis factor alpha (TNF-α), and interleukin 6 (IL-6) [61]. Likewise, individuals diagnosed with anxiety disorders also present with increased concentrations of inflammatory markers [62]. Given that adverse life changes in later adulthood cause chronic worry and stress, which lead to an increased inflammatory response (due to the dysregulation of the hypothalamic–pituitary–adrenal axis) [63], it is speculated that a higher SWL can decrease anxiety symptoms in the elderly by reducing inflammation through the suppression of an overactive immune system. Therefore, among elderly with a higher level of BMI, the neuroprotective effect of SWL is counteracted by the abnormal inflammatory response associated with obesity and the coexisting high dietary fat intake. This would negate the neuroprotective effect of SWL on anxiety symptoms in Chinese elderly with a higher BMI. However, in Chinese elderly with lower and average levels of BMI, BMI is discovered to facilitate less anxiety symptoms which may be partially due to the synergistic effects of both SWL and BMI on immune response regulation. While, these assumptions need to be investigated in future studies. To the best of our knowledge, this is the first study highlighting the moderator role of BMI in the association between SWL and anxiety symptoms. In addition to promoting a positive psychology programme, the interaction between psychological and physical factors influencing anxiety symptoms revealed in this study, suggests a multi-level intervention model for Chinese elderly during clinical practice. The multi-level model should include physical interventions with nutritional counselling and regular exercise to achieve physical fitness and to avoid overweight and obesity [64]. It should also include a psychological intervention using positive psychology to achieve subjective well-being.

In contrast to previous studies, we did not find any association between cognitive function and anxiety symptoms at subclinical levels in Chinese elderly. One reason for non-significant result may be due to the limited sample size which lacked statistical power to detect a significant effect. Another reason may be due to the cognitive instrument used in the present study which may not be sensitive enough to reflect the precise cognitive process. For example, in the study by Gulpers et al., the individual’s capabilities for attention, verbal memory, executive function, and information processing speed were measured using the Concept Shifting Test, the Visual Verbal Word Leaning Test, the Stroop Color Word Test, and the Letter Digit Substitution Test, respectively [29]. Although the HK-MoCA sufficiently evaluates global cognitive function and screens individuals with mild cognitive impairment, it has limitations in the accurate assessment of different cognitive domains. Recently, Feng et al. reported that worry caused by adverse life events is maintained and strengthened by an individual’s deficiencies in attention and interpretational memory (cognitive function), which can predict anxiety symptoms in the general population [56]. In contrast to this, we did not identify a mediating effect of cognitive function in the association between SWL and anxiety symptoms, which needs to be further investigated using more specific cognitive instruments in future studies.

There are several limitations of the present study. First, our findings cannot suggest a causal relationship because of the cross-sectional study design, although a strong association between SWL and anxiety symptoms and a significant moderating effect of BMI were identified in Chinese elderly. Future research in this area is recommended to verify our findings using a prospective longitudinal study design. Second, we used self-reported questionnaires to measure anxiety symptoms, which may yield responses affected by an individual’s honesty, memory, and social context. These factors can lead to incorrect or inaccurate responses, and subsequently increase the bias in our findings. Future research is recommended to supplement the present subjective questionnaires using objective structured clinical interviews, as the combined data collection approaches will avoid potential errors and provide more comprehensive information. Third, more specific cognitive instruments specifically measuring different cognitive domains should be used in future studies, instead of the general cognitive performance test used in the present study. Other cognitive factors, such as interpretation bias (i.e., interpretation of ambiguous situations in a negative way) should also be measured in future studies to better understand the underlying neuropathological mechanisms of the association between SWL and anxiety symptoms at subclinical levels. Fourth, this pilot study was only based in Hong Kong, a modern city in Southern China. Considering the economic level, living conditions, and cultural differences between Southern and Northern China, findings in this study cannot be representative of the overall elderly population in China. Future research should expand the present study by collecting samples of multiple provinces/cities of China to generalize the findings for a larger population of Chinese elderly.

In the present pilot study, we constructed a moderated mediation model to investigate the linking pathway between SWL and anxiety symptoms in a subclinical ageing population. The subclinical population has been found to share risk factors as well as underlying neuropathological mechanisms related with clinical diagnosed anxiety. For the first time, we demonstrated the moderating effect of BMI underpinning the association between SWL and anxiety symptoms among Chinese elderly in Hong Kong. Our findings show that life satisfaction could reduce anxiety symptoms among Chinese elderly as their BMI decreases (when BMI ranged between “mean-1SD” and “mean” of the population). The significant interaction between psychological and physical variables shown in the present study will be translated to promote multi-level psychological and physical interventions for ageing patients with anxiety during clinical practice.

## Data Availability

The dataset used and/or analyzed during the present study are available from the corresponding author on reasonable request.

## Abbreviations

BMI: Body Mass Index
TONI-4: Test of Nonverbal Intelligence, Fourth Edition
SWLS: Satisfaction with Life Scale
HK-MoCA: Hong Kong Version of Montreal Cognitive Assessment
HAM: Hamilton Anxiety Rating Scale
